# Determinants of gait independence after mechanical ventilation in the intensive care unit: a Japanese multicenter retrospective exploratory cohort study

**DOI:** 10.1186/s40560-019-0404-2

**Published:** 2019-11-27

**Authors:** Shinichi Watanabe, Toru Kotani, Shunsuke Taito, Kohei Ota, Kenzo Ishii, Mika Ono, Hajime Katsukawa, Ryo Kozu, Yasunari Morita, Ritsuro Arakawa, Shuichi Suzuki

**Affiliations:** 1 Department of Rehabilitation Medicine, National Hospital Organization, Nagoya Medical Center, 4-1-1 Sannomaru, Naka-ku, Nagoya, Aichi 460-0001 Japan; 2 Department of Intensive Care Medicine, School of Medicine, Showa University, 1-5-8 Hatanodai, Shinagawa-ku, Tokyo, 142-8666 Japan; 3 Division of Rehabilitation, Department of Clinical Practice and Support, Hiroshima University Hospital, Hiroshima, Japan; 4 Department of Emergency and Critical Care Medicine, Hiroshima University, 1-2-3, Kasumi, Minami-ku, Hiroshima, 734-8551 Japan; 5 Department of Anesthesia, Fukuyama City Hospital, 5-23-1, Zaou-tyo, Hukuyama, Hiroshima, 734-0971 Japan; 6 Department of Nursing, Nagoya University of Arts and Sciences, 4-1-1 Sannomaru, Naka-ku, Nagoya, Aichi 460-0001 Japan; 7 Japanese Society for Early Mobilization, 1-2-12 Kudankita, Tiyoda-ku, Tokyo, 102-0073 Japan; 8 Department of Cardiopulmonary Rehabilitation Science, Nagasaki University Graduate School of Biomedical Sciences, 1-7-1 Sakamoto, Nagasaki, 852-8520 Japan; 9 Department of Rehabilitation Medicine, Nagasaki University Hospital, 1-7-1 Sakamoto, Nagasaki, 852-8520 Japan; 10 Department of Critical Care Medicine, National Hospital Organization, Nagoya Medical Center, 4-1-1 Sannomaru, Naka-ku, Nagoya, Aichi 460-0001 Japan

**Keywords:** Gait independence, Mechanical ventilation, Intensive care unit-acquired weakness, Early mobilization

## Abstract

**Purpose:**

Gait independence is one of the most important factors related to returning home from the hospital for patients treated in the intensive care unit (ICU), but the factors affecting gait independence have not been clarified. This study aimed to determine the factors affecting gait independence at hospital discharge using a standardized early mobilization protocol that was shared by participating hospitals.

**Materials and methods:**

Patients who entered the ICU from January 2017 to March 2018 were screened. The exclusion criteria were mechanical ventilation < 48 hours, age < 18, loss of gait independence before hospitalization, being treated for neurological issues, unrecoverable disease, unavailability of continuous data, and death during ICU stay. Basic attributes, such as age, ICU length of stay, information on early mobilization while in the ICU, Medical Research Council (MRC) sum-score at ICU discharge, incidence of ICU-acquired weakness (ICU-AW) and delirium, and the degree of gait independence at hospital discharge, were collected. Gait independence was determined using a mobility scale of the Barthel Index, and the factors that impaired gait independence at hospital discharge were investigated using a Cox proportional hazard regression analysis.

**Results:**

One hundred thirty-two patients were analyzed. In the univariate analysis, age, APACHE II score, duration of mechanical ventilation, ICU length of stay, incidence of delirium, and MRC sum-score at ICU discharge were extracted as significant. In the multivariate analysis, age (*p* = 0.014), MRC sum-score < 48 (*p* = 0.021), and delirium at discharge from ICU (*p* < 0.0001) were extracted as significant variables.

**Conclusions:**

We found that age and incidence of ICU-AW and delirium were significantly related to impaired gait independence at hospital discharge.

## Introduction

Advances in intensive care have led to a paradigm shift of the treatment goal from “saving life” to “returning home with full physical and mental recovery.” Patients treated with mechanical ventilation and sedation in the intensive care unit (ICU) face increased risks of functional disorders and impaired mobility as a result of disuse syndrome [1] and require prolonged rehabilitation [2]. Several studies have shown that early mobilization provides better quality of life after ICU discharge [3–5]. Contrarily, muscle weakness developed during hospitalization, the so-called ICU-acquired weakness (AW), and delirium are factors that reduce the quality of life after discharge and delay resocialization. In addition, it is reported that the incidence of ICU-AW and delirium not only prolonged the duration of mechanical ventilation and length of ICU stay, but also impaired general activities of daily living including gait and cognitive function [6–9]. The American Thoracic Society and American College of Chest Physicians published clinical practice guidelines that recommend interventions to achieve early mobilization in patients who expected more than 24 h of mechanical ventilation [10]. Early mobilization carried out with a clear protocol provided functional independence, including gait, as a goal of ICU rehabilitation [9, 11, 12].

However, effective ICU rehabilitation leading to improved home discharge rates is still unclear. Gait independence is considered one of the most important factors related to returning home for patients treated in the ICU [5, 13]. Although effective early mobilization on achieving gait independence has been reported in a few studies [5, 14–17], the factors that affect gait independence at hospital discharge have not been investigated. We established a multicenter research group 3 years ago to explore the effective early mobilization protocol. We shared the previously published standardized protocol among the eight participating hospitals. We found, however, a considerable number of patients had impaired gait independence at hospital discharge. To further improve early mobilization protocol (Appendix 1), it is essential to determine risk factors responsible for losing gait independence and to provide countermeasures.

The purpose of the study is to assess the data of the participating hospitals retrospectively and seek the potential factors associated with gait dependence at hospital discharge. We hypothesized that incidence of ICU-AW and delirium may be negatively associated with gait independence as demonstrated above.

## Methods

### Study design and subject

We reviewed medical records of the patients treated in the ICU between January 2017 and March 2018 in eight tertiary hospitals in Japan. Patients who were mechanically ventilated for equal to or more than 48 h in the ICU were screened. Patients with ages less than 18 years, loss of gait independence before hospitalization [18], being treated for neurological issues, unrecoverable disease, unavailability of continuous data, and death during ICU stay were excluded from the study. Patients requiring wheelchair or other gait assistance except a walking stick before admission were excluded.

The number of ICU beds in each hospital is shown in Appendix 2. Protocols for sedation, analgesia, and weaning were not shared. However, the protocol for rehabilitation used in the previous study [19] was shared in the participating hospitals, and ICU staff members were trained and fully compliant with the protocol. The start and cancelation criteria of the protocol are shown in Appendix 1. Before starting the current study, the participating hospitals had a 6-month preparation period to carry out the early mobilization study protocol and data collection for the standardization of the quantity (frequency) and quality of intervention. All patients were provided the usual rehabilitation sessions on a continuous basis only by physical or occupational therapists after ICU discharge.

Each participating hospital obtained approval of the study by the respective ethics committee (the Nagoya Medical Center Hospital Institutional Review Board; approval number: 2018-19).

### Date collection

We collected detailed information at initial hospitalization and ICU discharge. We also collected data regarding independent gait ability upon hospital discharge. All data were obtained as a usual clinical practice.

Information at admission included age, sex, body weight, body mass index (BMI), main cause of ICU admission, Charlson’s Comorbidity Index (CCI) [20], Acute Physiology and Chronic Health Evaluation (APACHE) II score [21], and the Sequential Organ Failure Assessment (SOFA) score [22]. Data during ICU stay included the time to first rehabilitation assessment, duration of mechanical ventilation, time to first out-of-bed mobilization, and highest score achieved on the ICU-mobility scale (IMS) [23]. We also investigated the incidence of adverse events during rehabilitation, such as cardiopulmonary arrest, fall to knees or the ground, inadvertent removal of medical devices, desaturation (< 90%) or more than 10% decrease from the baseline, bradypnea (< 5 breaths/min), tachypnea (> 40 breaths/min), bradycardia (< 40 beats/min), tachycardia (> 130 beats/min), hypotension (systolic blood pressure [SBP] < 80 mmHg), hypertension (SBP > 200 mmHg), and newly occurring arrhythmia. At ICU discharge, we collected incidence of ICU-acquired weakness (ICU-AW) and delirium, respectively. As mentioned above, early mobilization was performed according to the previous protocol [19] consisted with five session levels (see Appendix 1). We investigated the number of times levels 3, 4, and 5 were achieved, and total number of times levels higher than level 2 were achieved. We calculated ICU length of stay at ICU discharge, and hospital length of stay and ratio of home discharge at hospital discharge.

The IMS provides a quick and simple bedside method of measuring the mobility of a critically ill patient. As functional endpoints in studies of rehabilitation in the ICU, the IMS provides a sensitive 11-point ordinal scale, ranging from nothing (lying/passive exercises in bed, score of 0) to independent ambulation (score of 10). ICU-AW was evaluated using Medical Research Council (MRC) sum-score by the responsible physical therapist, and a value of less than 48 was defined as having developed an ICU-AW [24, 25]. The cooperation-level assessment was carried out, and muscle strength tests were only performed when the subject correctly answered the five questions [26]. For the assessment of delirium, either the delirium screening tool of the Confusion Assessment Method for the Intensive Care Unit (CAM-ICU) [27] or the Intensive Care Delirium Screening Checklist (ICDSC) [28] was used according to the usual practice of each participating hospital. Outcomes other than home discharge included transfers to rehabilitation hospitals and to nursing homes.

Patients who could walk 45 m or more with or without braces were determined as gait independent. We also used mobility scale of the Barthel Index (BI) to quantitatively assess gait independence [18, 29]. BI is the most widely used ADL scale, and its reliability and relevance have been recognized [30]. Because we previously determined BI was an effective mobility parameter to assess the achievement of gait independence [31], we used this parameter in the current study. BI was measured at ICU and hospital discharge.

### Statistical analysis

We compared the basic attributes and rehabilitation progress factors expressed by the median (interquartile range) or the number of cases (%) in the data in both groups. The Mann-Whitney test was used for intergroup comparisons of the continuous and ordinal variables of each item, and the intergroup comparison of the nominal variables was examined using the *χ*^2^ test. For the multivariate analysis, we used gait independence at discharge as the dependent variable, and the explanatory variables were the items other than the variables of measurement at discharge. A Cox proportional hazards regression analysis was used, in which all items with *p* < 0.05 in a univariate analysis were input into the multivariate model. We assessed the probability of gait independence at hospital discharge as a function of the interval from ICU discharge to hospital discharge using the Kaplan-Meier curve. JMP version 13.0 (SAS Institute, Cary, NC, USA) was used for statistical analysis. The significance level was less than 5%.

## Results

**Fig. 1:**
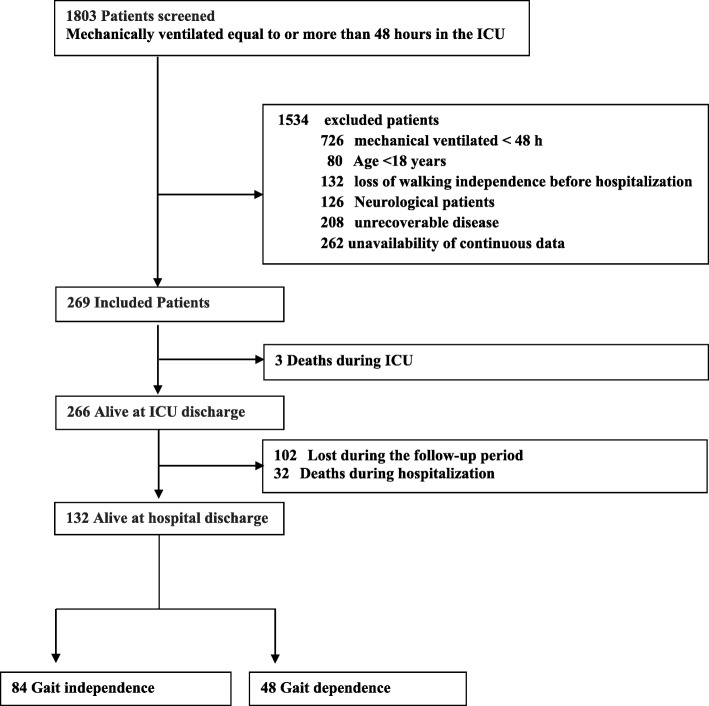
Flow chart of patient selection process

During the study period, 1803 patients were screened and 269 patients were included in the study. Thirty-five died during hospitalization, and 102 patients were lost during follow-up period. Finally, 132 patients were discharged. MRC sum-score at ICU discharge was measured in all these patients (Fig. 1). Independent gait at hospital discharge was observed in 84 patients (independence group), but not in 48 patients (dependence group). Table 1 shows the demographic data of the patients in the total, independence, and dependence group. In the comparison of both groups, there was a significant difference in age (*p* < .0001).

**Table 1:** Baseline characteristics and comparisons between study groups

	Total (*n* = 132)	Gait independence (*n* = 84)	Gait dependence (*n* = 48)	*p* value
Age (years)	69 [60–78]	67 [54–75]	76 [67–82]	< .0001
Male sex (%)	84 (63.6)	56 (66.7)	28 (58.3)	0.340
Weight (kg)	57 [50–70]	58 [51–69]	57 [49–73]	0.406
Body mass index (kg/m^2^)	22 [20–26]	23 [20–26]	21.7 [19–27]	0.609
Charlson’s Comorbidity Index	2 [1–3]	2 [1–3]	1 [1–3]	0.612
Main cause of ICU admission (%)				
Respiratory (including pneumonia)	25 (18.9)	15 (17.8)	10 (21.3)	0.457
Cardiovascular	23 (17.4)	15 (17.8)	8 (16.6)	
Gastrointestinal	20 (15.2)	13 (15.5)	7 (14.5)	
Trauma	18 (13.6)	10 (11.9)	8 (16.6)	
Sepsis, nonpulmonary	17 (12.9)	12 (14.3)	5 (10.3)	
Other	29 (22.0)	19 (22.7)	10 (20.7)	
APACHE II score	24 [20–29]	23 [18–27]	24 [20–30]	0.129
SOFA score at ICU admission	9 [7–11]	9 [7–11]	9 [8–12]	0.217

Median [25th–75th percentile] or the number of patients (percentage). Independent-sample Mann-Whitney *U* test or *χ*^2^ test. *APACHE II* Acute Physiology and Chronic Health Evaluation, *SOFA* Sequential Organ Failure Assessment

Table 2 shows a comparison of clinical outcomes during hospitalization. The independence group showed a significant decrease in the length of ICU stays (*p* = 0.025), MRC sum-score < 48, and delirium at ICU discharge (*p* < .0001) compared to the dependence group. The home discharge ratio in the independence group was significantly higher compared to that in the dependence group (*p* < .0001). There were no significant differences in the duration of mechanical ventilation; the time to first out-of-bed mobilization; the mobility status, such as the highest IMS; the hospital length of stay; or the incidence of adverse events. Sixty-six out of 132 patients (50%) had ICU-AW at ICU discharge. Supplemental data are shown in Appendix 3.

**Table 2:** Comparison of the clinical outcomes between study groups

	Total (*n* = 132)	Gait independence (*n* = 84)	Gait dependence (*n* = 48)	*p* value
ICU and hospital outcome				
Time to first rehabilitation (day)	2 [2–4]	2 [2–4]	3 [2–4]	0.906
Duration of mechanical ventilation (day)	5 [3–7]	5 [3–7]	5 [4–8]	0.211
Time to first out-of-bed mobilization (day)	6 [4–9]	6 [4–10]	6 [4–9]	0.151
Highest reach IMS at ICU entry	4 [3–5]	3 [3–5]	3 [3–6]	0.089
Delirium, *n* (%)	51 (38.6)	23 (27.7)	28 (58.3)	< .0001
MRC sum-score at ICU discharge	45 [36–48]	48 [36–54]	36 [28–48]	< .0001
ICU-AW at ICU discharge, *n* (%)	66 (50.0)	36 (42.9)	30 (62.5)	< .0001
ICU length of stay (day)	8 [5–11]	7 [5–11]	8 [6–12]	0.025
Hospital length of stay (day)	40 [22–59]	41 [21–61]	40 [22–57]	0.909
Discharge to home, *n* (%)	81 (61.4)	60 (71.4)	21 (43.8)	< .0001
Early mobilization levels, session (%)				
Level 3	121 (70.5)	76 (69.1)	44 (73.3)	0.560
Level 4	41 (24.1)	27 (24.5)	14 (23.3)	0.859
Level 5	9 (5.4)	7 (6.4)	2 (3.4)	0.495
Total session for levels 3 to 5	170	110	60	–
Adverse event during ICU rehabilitation, *n* (%)				
Cardiopulmonary arrest	0 (0)	0 (0)	0 (0)	–
Fall to knees or ground	0 (0)	0 (0)	0 (0)	–
Inadvertent removal of medical devices	0 (0)	0 (0)	0 (0)	–
Desaturation	4 (1.8)	1 (0.9)	3 (6.0)	0.255
Tachypnea or bradypnea	1 (0.4)	0 (0)	1 (2.0)	–
Tachycardia or bradycardia	4 (1.8)	3 (2.7)	0 (0)	–
Hypertension or hypotension	10 (4.4)	4 (3.6)	3 (6.0)	0.512
New arrhythmia	0 (0)	0 (0)	0 (0)	–

Median [25th–75th percentile] or the number of patients (percentage). Independent-sample Mann-Whitney *U* test or *χ*^2^ test. *ICU* intensive care unit, *IMS* ICU-mobility scale, *MRC* Medical Research Council, *ICU-AW* ICU-acquired weakness, *BI* Barthel Index

**Fig. 2:**
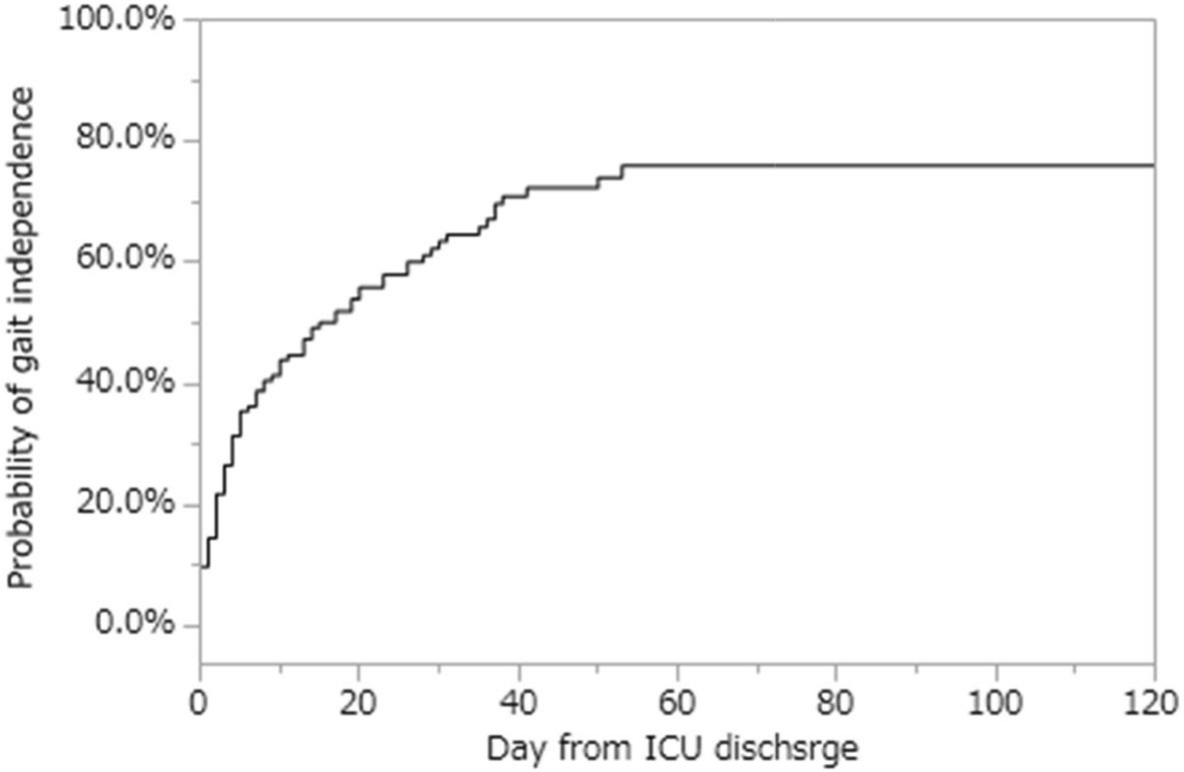
The Kaplan-Meier curve of gait independently at hospital discharge

Table 3 shows the results of univariate and multivariate analysis performed to identify potential factors for gait independence. In the univariate analysis, age, APACHE II score, ICU length of stay, incidence of delirium, and MRC sum-score at ICU discharge were extracted as significant. In the multivariate analysis, age, incidence of MRC sum-score < 48, and delirium at discharge from ICU were extracted as significant variables. The Kaplan-Meier curve to show the probability of gait independence from ICU discharge is presented in Fig. 2.

**Table 3:** Factors affecting gait independence at hospital discharge

Baseline characteristics	Univariate analysis (*n* = 132)		Multivariate analysis (*n* = 132)	
	HR (95% CI)	*p* value	HR (95% CI)	*p* value
Age (1 year)	1.02 (1.01–1.04)	0.008	1.02 (1.01–1.04)	0.008
Male	1.10 (0.70–1.76)	0.670		
Weight (1 kg)	0.99 (0.98–1.01)	0.141		
Body mass index (1 kg/m^2^)	0.98 (0.95–1.03)	0.101		
Charlson’s Comorbidity Index (× 1 point)	1.01 (0.97–1.14)	0.235		
APACHE II score (1 point)	1.05 (1.02–1.08)	0.011	1.02 (0.95–1.03)	0.192
SOFA score at ICU admission (1 point)	1.06 (0.97–1.14)	0.210		
Time to first rehabilitation assessment (1 day)	1.00 (0.91–1.08)	0.968		
Time to first out-of-bed mobilization (1 day)	1.01 (0.99–1.06)	0.281		
Duration of mechanical ventilation (1 day)	1.03 (1.01–1.07)	0.040		
ICU length of stay (1 day)	1.05 (1.02–1.09)	0.005	1.03 (0.93–1.09)	0.167
Highest reach IMS at ICU entry	0.92 (0.83–1.03)	0.095		
Delirium	2.04 (1.27–3.38)	< 0.001	1.49 (1.05–2.42)	0.033
MRC sum-score at ICU discharge (1 point)	0.94 (0.91–0.96)	< 0.001		
MRC sum-score < 48 at ICU discharge	2.89 (1.86–4.55)	< .0001	2.16 (1.32–338)	< .0001

*APACHE* II Acute Physiology and Chronic Health Evaluation, *SOFA* Sequential Organ Failure Assessment, *MRC* Medical Research Council, *HR* = hazard ratio

## Discussion

In this study, we retrospectively investigated the factors affecting gait independence at hospital discharge in the ICUs of eight different hospitals sharing the early mobilization protocol. To correctly use the protocol among the hospitals, we set up a preparation period prior to the study. We confirmed ICU-AW developed in half of the patients and was significantly related to gait independence at hospital discharge as well as age and delirium.

Previous studies have reported disease severity, complications, and duration of mechanical ventilation were associated with gait independence at hospital discharge [32, 33]. Another study has reported that the main risk factors for ICU-AW include high severity of illness upon admission, sepsis, multiple organ failure, prolonged immobilization, hyperglycemia, and age [34]. Therefore, we expected disease severity and duration of mechanical ventilation to be a determinant of gait independence. However, no physiological severity parameters, except age, were included in the results. One possible reason is that aforementioned studies were conducted without the standardized early mobilization protocol. It is suggested that the application of an appropriate protocol for early mobilization is key for the contribution of disease severity and duration of mechanical ventilation to gait independence.

Both ICU-AW and delirium affect not only mortality but also health-related quality of life and increase ICU length of stay [35–39]. Deep sedation is associated with ICU-AW and delirium [40, 41]. Additionally, there is considerable evidence that early mobilization can decrease the incidence of ICU-AW and delirium [40–42]. In this study, however, we started mobilization sessions as early as the third ICU-day according to our early mobilization protocol and found that ICU-AW and delirium occurred in 50% and 35.4% of the patients at ICU discharge, respectively. This incidence was comparable to previous studies [43, 44]. In the ICU setting, pain, discomfort, delirium, immobility, and sleep are problems, and it is recommended to carry out early mobilization under appropriate analgesia and sedation management [45]. A single-center randomized controlled study reported the failure of early mobilization in the reduction of ICU stay, ventilator days, or preservation of muscle strength [46], and the lack of a clear sedation protocol possibly contributed to the results [47]. The lack of a sedation protocol caused a similar situation in our study, another consequence of early mobilization. To increase MRC sum-score at ICU discharge, it is suggested to introduce new rehabilitation program which could be performed on the bed during levels 1 and 2 such as electrical muscle stimulation [48] because it can be implemented even when the patient can afford passive exercise only. To reduce delirium, it is suggested that a comprehensive management system that includes uniform protocols of sedation, analgesics, and mechanical ventilation withdrawal is shared as a standard care among the participating facilities.

The average ICU length of stay was 8 days. Consequently, out-of-bed mobilization could be provided on average only once or twice during the ICU stay. On the other hand, the median length of hospitalization was 40 days. Because the criteria for hospital discharge are different among the facilities, this factor could have some influence on gait independence. However, there was no significant difference between hospital length of stay and gait independence. Additionally, there was no significant difference in the distribution of hospital days and the ratio of gait independence. Taken together, the association of gait independence with hospital length of stay was limited. On the other hand, the home discharge rate was significantly higher in the independence group. The factors age, decreasing delirium, and MRC sum-score more than 48 will become important indicators as a major goal of rehabilitation.

This study has several limitations. First, we shared a well-defined protocol for early mobilization but not for sedation, analgesia, and weaning from mechanical ventilation. We could not perfectly collect the data on medication [49], such as the type of muscle relaxant or vasopressor prescribed, the cumulative dose used, and the use of glucocorticoids, which are associated with ICU-AW. Also, we did not investigate the effects of the invasive treatments in the ICU that can make the patient bedridden (continuous renal replacement therapy, veno-venous extracorporeal membrane oxygenation, intra-aortic balloon pumping, etc.) and cognitive disorders. Although the influence of lacking those data is not negligible, we believe that increasing MRC sum-score and decreasing the length of ICU stay are important factors affecting gait independence at hospital discharge. Second, the frequency and intensity of rehabilitation therapies provided after ICU discharge were not investigated. Finally, the first out-of-bed mobilization was performed on the sixth ICU day in our study, which is 1 day longer than the ventilation day, likely because out-of-bed mobilization was started after extubation in most patients. Lacking a shared protocol for weaning from mechanical ventilation caused delays that may contribute to decreasing MRC sum-score.

## Conclusions

We analyzed factors contributing to gait independence at hospital discharge in mechanically ventilated patients in the eight ICUs sharing a uniform mobilization protocol. We found muscle weakness (ICU-AW) at ICU discharge, age, and incidence of delirium as significant determinants. Further study is warranted to clarify whether reducing ICU-AW and incidence of delirium improves gait independence.

## Data Availability

The datasets used and/or analyzed during the current study are available from the corresponding author on reasonable request.

## Appendix 1

**Table 4:** Multihospital common early mobilization protocol

Level 1 respiratory (RASS − 5 approximately to – 3)	Level 2 HOB (RASS ≥ − 3)	Level 3 sitting (RASS ≥ − 1)	Level 4 standing (RASS ≥ 0)	Level 5 gait (RASS ≥ 0)
Physical therapy □ Passive ROM exercise □ Respiratory physical therapy	Physical therapy □ Positioning □ Passive ROM exercise □ Active ROM exercise □ Respiratory physical therapy □ Continuous lateral rotation therapy	Physical therapy □ Positioning □ Passive ROM exercise □ Active ROM exercise □ Sitting at side of bed □ Rising from the supine position	Physical therapy □ Positioning □ Passive ROM exercise □ Active ROM exercise □ Standing at side of bed □ Stand and pivot to a chair	Physical therapy □ Positioning □ Passive ROM exercise □ Active ROM exercise □ Walk with assistance □ Walk independently
Positioning □ Posture change □ HOB ≤ 45 degrees	Positioning □ Posture change □ HOB ≥ 60	Positioning □ Posture change □ HOB ≥ 60	Positioning □ Posture change □ HOB ≥ 60	Positioning □ Posture change □ HOB ≥ 60
Step up criterion □ Oxygenation/hemodynamic stability □ Can withstand posture change □ Can withstand HOB ≤ 45 degrees	Step up criterion □ Can withstand supplementary motion of physical therapy □ Can withstand HOB ≤ 60 degrees □ Anti-gravity movement possible	Step up criterion □ Can endure the active movement of physical therapy □ Can withstand HOB ≤ 60 degrees □ Can withstand sitting at side of bed	Step up criterion □ All exercise can be carried out □ Can withstand partial weight standing	Step up criterion □ Increase walking distance gradually
Step up criterion to level 3 or higher are defined as: RASS − 1 to + 2, BPS ≤ 3 or NRS ≤ 5, SpO_2_ ≥ 91%, FIO_2_ > 0.6, respiratory rate < 30 times/min, systolic blood pressure 90 to 180 mmHg, mean blood pressure 65 to 110 mmHg, heart rate 60 to 120 times/min, there were no new arrhythmias, no additional administration of vasopressors, no bleeding, no wound with the possibility of separation, and no unstable fracture.				

*RASS* Richmond Agitation-Sedation Scale, *ROM* range of motion, *HOB* head of bed, *BPS* behavioral pain scale, *NRS* numeric rating scale

## Appendix 2

**Table 5:** Background of research participation hospitals

	Number of bed	ICU system	Number of ICU bed	Patient to nurse ratio	Dedicated physiotherapist	Sedation protocol
A	740	Mandatory	26	2:1	Yes	Yes
B	740	Mandatory	8	2:1	Yes	No
C	592	Closed	12	1:1	No	No
D	500	Closed	6	2:1	No	No
E	464	Elective	32	2:1	Yes	No
F	415	Open	8	2:1	No	No
G	376	Mandatory	10	2:1	No	No
H	464	Open	10	2:1	No	No

Closed, ICU physician decides therapeutic policy; Mandatory, ICU physician is involved in deciding treatment policy; Elective, ICU physician is mainly involved as a consultant; Open, each department of medicine determines its own treatment policy

## Appendix 3

**Table 6:** Relationship between gait independence group and hospital length of stay

Hospital length of stay	0–25 days	26–50 days	51–75 days	76–100 days	100 days more	*p* value
Gait independence (*n* = 84)	26 (31)	32 (38)	16 (20)	4 (5)	6 (6)	0.928
Gait dependence (*n* = 48)	16 (33)	15 (31)	10 (21)	2 (4)	5 (11)	

The number of patients (percentage). Independent-sample *χ*^2^ test

## Abbreviations

BI: Barthel Index
BMI: Body mass index
BPS: Behavioral pain scale
HOB: Head of bed
IMS: ICU-mobility scale
MHAQ: Modified Health Assessment Questionnaire
MRC: Medical Research Council
ROM: Range of motion
SOFA: Sequential Organ Failure Assessment
